# Use of almitrine in spontaneously breathing patients with COVID-19 treated with high-flow nasal cannula oxygen therapy and with persistent hypoxemia

**DOI:** 10.1186/s12931-022-02308-y

**Published:** 2023-01-05

**Authors:** Clément Saccheri, Lucas Morand, Marie Juston, Denis Doyen, Hervé Hyvernat, Romain Lombardi, Raphaël Devanlay, Émilie Panicucci, Jean Dellamonica, Mathieu Jozwiak

**Affiliations:** 1 Service de Médecine Intensive Réanimation, Centre Hospitalier Universitaire de Nice, Hôpital L’Archet 1, 151 Rue Saint Antoine de Ginestière, 06200 Nice, France; 2 Équipe 2 CARRES, UR2CA - Unité de Recherche Clinique Côte d’Azur, Université Côte d’Azur, Nice, France

**Keywords:** Acute respiratory distress syndrome, Awake prone positioning, COVID-19, Mechanical ventilation, Oxygenation

## Abstract

**Background:**

Almitrine, a selective pulmonary vasoconstrictor in hypoxic area, improves oxygenation in mechanically ventilated patients with COVID-19 but its effects in spontaneously breathing patients with COVID-19 remain to be determined.

**Methods:**

We prospectively studied the effects of almitrine (16 µg/kg/min over 30 min followed by continuous administration in responders only) in 62 patients (66% of male, 63 [53–69] years old) with COVID-19 treated with high-flow nasal cannula oxygen therapy (HFNO) and with persistent hypoxemia, defined as a PaO_2_/FiO_2_ ratio < 100 with FiO_2_ > 80% after a single awake prone positioning session. Patients with an increase in PaO_2_/FiO_2_ ratio > 20% were considered as responders.

**Results:**

Overall, almitrine increased the PaO_2_/FiO_2_ ratio by 50% (p < 0.01), decreased the partial arterial pressure of carbon dioxide by 7% (p = 0.01) whereas the respiratory rate remained unchanged and 46 (74%) patients were responders. No patient experienced right ventricular dysfunction or acute cor pulmonale. The proportion of responders was similar regardless of the CT-Scan radiological pattern: 71% for the pattern with predominant ground-glass opacities and 76% for the pattern with predominant consolidations (p = 0.65). Responders had lower intubation rate (33 *vs.* 88%, p < 0.01), higher ventilator-free days at 28-day (28 [20–28 ] *vs. *19 [2–24] days, p < 0.01) and shorter ICU length of stay (5 [3–10] *vs.*12 [7–30] days, p < 0.01) than non-responders.

**Conclusions:**

Almitrine could be an interesting therapy in spontaneously breathing patients with COVID-19 treated with HFNO and with persistent hypoxemia, given its effects on oxygenation without serious adverse effects regardless of the CT-Scan pattern, and potentially on intubation rate. These preliminary results need to be confirmed by further randomized studies.

**Supplementary Information:**

The online version contains supplementary material available at 10.1186/s12931-022-02308-y.

## Background

Since December 2019, a worldwide pandemic of Coronavirus disease (COVID-19) secondary to the emerging coronavirus SARS-CoV-2 was observed from China. Although most patients are asymptomatic or developed a non-severe form of pneumoniae, 1–3% of patients will develop a hypoxemic acute respiratory failure, requiring admission to intensive care unit (ICU) [1–3].

It has been suggested that the physiopathology of hypoxemia in patients with pneumonia related to SARS-CoV-2 might mainly resulted from a more marked impairment of the hypoxic pulmonary vasoconstriction than in non-COVID-19 patients [4], leading in the most severe patients to untypical acute respiratory distress syndrome (ARDS) with a marked hypoxemia secondary to large intrapulmonary shunt but a preserved lung compliance [5, 6]. Thus, the administration of almitrine, a selective pulmonary vasoconstrictor in hypoxic area, may be interesting in these patients [4]. Some retrospective or pilot studies have reported that almitrine administration as rescue therapy improved oxygenation of mechanically ventilated patients with ARDS related to SARS-CoV-2 pneumonia [7–10]. However, its effects in spontaneously breathing patients with severe pneumonia related to SARS-CoV-2 pneumonia remain to be determined.

The first aim of this study was to assess the effects of almitrine on oxygenation in spontaneously breathing patients with severe pneumonia related to SARS-CoV-2 pneumonia treated with high-flow nasal cannula oxygen therapy (HFNO) and with persistent hypoxemia. The second aim was to assess the effects of almitrine on oxygenation according to the thoracic CT-Scan patterns.

## Methods

This prospective and observational study was conducted in the 13-bed intensive care unit (ICU) of the University Hospital of Nice and was approved by the Ethics committee of the Société de Réanimation de Langue Française (CE SRLF 22–007). Informed consent was waived but all patients or next of kin were informed about the study. The study complied with the Strengthening the Reporting of Observational Studies in Epidemiology (STROBE) statement guidelines (Additional file 1) [11].

We included all patients over 18 years old admitted to ICU for SARS-CoV-2 pneumonia treated with HFNO and with persistent hypoxemia from January 2021 (start of the use of almitrine in our ICU for patients with COVID-19) to January 2022. Persistent hypoxemia was defined by a partial arterial pressure of oxygen over inspired oxygen fraction (PaO_2_/FiO_2_) ratio < 100 with FiO_2_ > 80% after a single awake prone positioning session. All patients had a positive real-time reverse transcriptase-polymerase chain reaction assay for SARS-CoV-2 in nasal swabs.

Exclusion criteria were (i) the need for immediate intubation because of respiratory, hemodynamic or neurological failure, (ii) contraindication to almitrine (acute liver failure, lactic acidosis, right ventricular failure, acute cor pulmonale, pregnancy) [12], (iii) patients without thoracic CT-Scan, (iv) patients with a decision to withdraw life-sustaining therapy, including do-not-intubate orders, and (v) patients with poor echogenicity, defined as the inability to correctly align the Doppler beam to obtain reliable Doppler measurements and/or to correctly delineate the endocardium for measuring the left and right ventricular end-diastolic area (LVEDA and RVEDA).

### Ventilatory management and respiratory measurements

HFNO (Optiflow^®^, Fisher and Paykel, Healthcare) was the first-line ventilatory support. The gas flow rate was set at 60 L/min and FiO_2_ was adjusted to maintain pulse oximetry (SpO_2_) ≥ 92% and not higher than 96% [13]. The ROX index was calculated just before and after administration of almitrine as follows: ratio of SpO_2_/FiO_2_ over the respiratory rate [14]. Non-invasive ventilation was always used as a second-line ventilatory support and was performed with an ICU ventilator (CARESCAPE R860; GE Healthcare, Chicago, IL, United States).

The indication of intubation was left at the discretion of the attending physician based on the intubation criteria used in our ICU during the study period: (i) respiratory rate > 40 breaths/min, (ii) a SpO_2_ < 90% for more than five minutes, (iii) occurrence or lack of improvement of signs of high respiratory-muscle workload, (iv) copious tracheal secretions, and/or (v) respiratory acidosis with a pH < 7.35 [15]. All patients were mechanically ventilated in volume assist-controlled mode and placed in 45-degree semi-recumbent position. Neuromuscular blocker agents and prone positioning were used according to current recommendations in non-COVID-19 patients with ARDS [16].

### Thoracic CT-Scan analysis

A thoracic CT-Scan (Lightspeed Ultra 8, GE Medical Systems, Milwaukee, WI) was performed prior to ICU admission in all patients. CT-Scan analysis was performed offline, by a 10-year experienced radiologist specialized in thoracic imaging. The severity of CT-Scan abnormalities was assessed by the radiologist according to the extent of ground-glass opacities and consolidations as a percentage of the total lung parenchyma: minimal: < 10%, moderate: 10–25%, extensive: 25–50%, severe: 50–75 and critical: > 75% [17]. Two main radiological patterns were identified: predominant ground-glass opacities and predominant consolidations [18]. The number of lung areas with organizing pneumonia was reported with a score ranging from 0 to 6 points, with 1 point for each of the six lung areas with the abnormality [17]. The same scoring system was used to report the number of lung areas with vascular enlargement sign, described as an unusual dilation of pulmonary vessels around and within the lesions in COVID-19 patients, which might be related to the congestion of alveolar septal capillaries [19, 20].

### Study protocol

A session of awake prone positioning was initially performed in all patients treated with HFNO and patients were asked to lie in prone position for as long as possible [21]. After this single awake prone positioning session, all patients with persistent hypoxemia received an almitrine bolus (16 µg/kg/min over 30 min) [22]. Immediately before and at the end of the almitrine bolus, respiratory, hemodynamic, echocardiographic and oxygenation variables were recorded (Additional File 2: Fig. S1). All other therapeutics remained unchanged during the study period. Patients with an increase in PaO_2_/FiO_2_ ratio > 20% after the almitrine bolus were considered responders [22]. In responders, administration of almitrine (16 µg/kg/min) was continued with twofold dose reduction every four hours if the PaO_2_/FiO_2_ ratio remained > 100, with a maximum administration time of 200 h [22]. Transthoracic echocardiographic examinations were performed daily until almitrine discontinuation to monitor hemodynamic adverse effects of almitrine (Additional file 2: Fig. S1). According to national guidelines during the study period, all patients received dexamethasone [23] and Tocilizumab was administered to all patients treated with HFNO with a C-reactive protein > 75 mg/L and worsening respiratory failure despite corticosteroids administration within the first 72 h after ICU admission [24].

### Data collection and endpoints

Patient characteristics, clinical, biological, echocardiographic, and radiological variables, therapeutics as well as ICU clinical outcomes were collected and analyzed. Both CT-scan analysis and echocardiographic measurements were analyzed offline, blinded to the patients’ response to almitrine. The respiratory comfort of patients was assessed by using the 100-mm visual analogue comfort scale [25].

The primary endpoint was the proportion of responders. Secondary endpoints were the proportion of responders according to the different thoracic CT-Scan patterns, the proportion of adverse effects of almitrine (RV systolic dysfunction, occurrence of acute cor pulmonale, occurrence of venous toxicity) the intubation rate, the number of ventilator-free days at 28-day, the ICU length of stay and the mortality rates in ICU, at 28-day and 90-day. For the calculation of ventilator-free days, one point was assigned for each day from the administration of almitrine bolus to 28-day that a patient was both alive and without invasive mechanical ventilation, and zero value was assigned to patients who died before day 28.

### Statistical analysis

Based on a PaO_2_/FiO_2_ ratio ranging from 80 to 100 mmHg in patients with COVID-19 before almitrine administration [7, 9, 10], we planned to include 60 patients to show an almitrine-induced clinically relevant increase in PaO_2_/FiO_2_ ratio of 20% [22] with a α risk of 0.05 and a power of 90%.

Normal distribution of continuous data was assessed using the Kolmogorov–Smirnov test. Continuous variables were expressed as median [interquartile range] and categorical variables as numbers (percentages). Between groups comparisons were performed by Student or Mann–Whitney tests for continuous variables and by Pearson’s Chi-square or Fisher exact tests for categorical variables. Within groups comparisons were performed by paired Student or Wilcoxon tests for continuous variables and by Mc Nemar or Fisher exact tests for categorical variables. To assess the ability of ROX index to predict response to almitrine, receiver operating characteristic curve with 95% confidence interval (CI) was generated.

Statistical analysis was performed with R 3.1.1 (R foundation for Statistical Computing Vienna, Austria). All tests were two-sided and a p-value < 0.05 was considered statistically significant.

## Results

### Study population

**Fig. 1:**
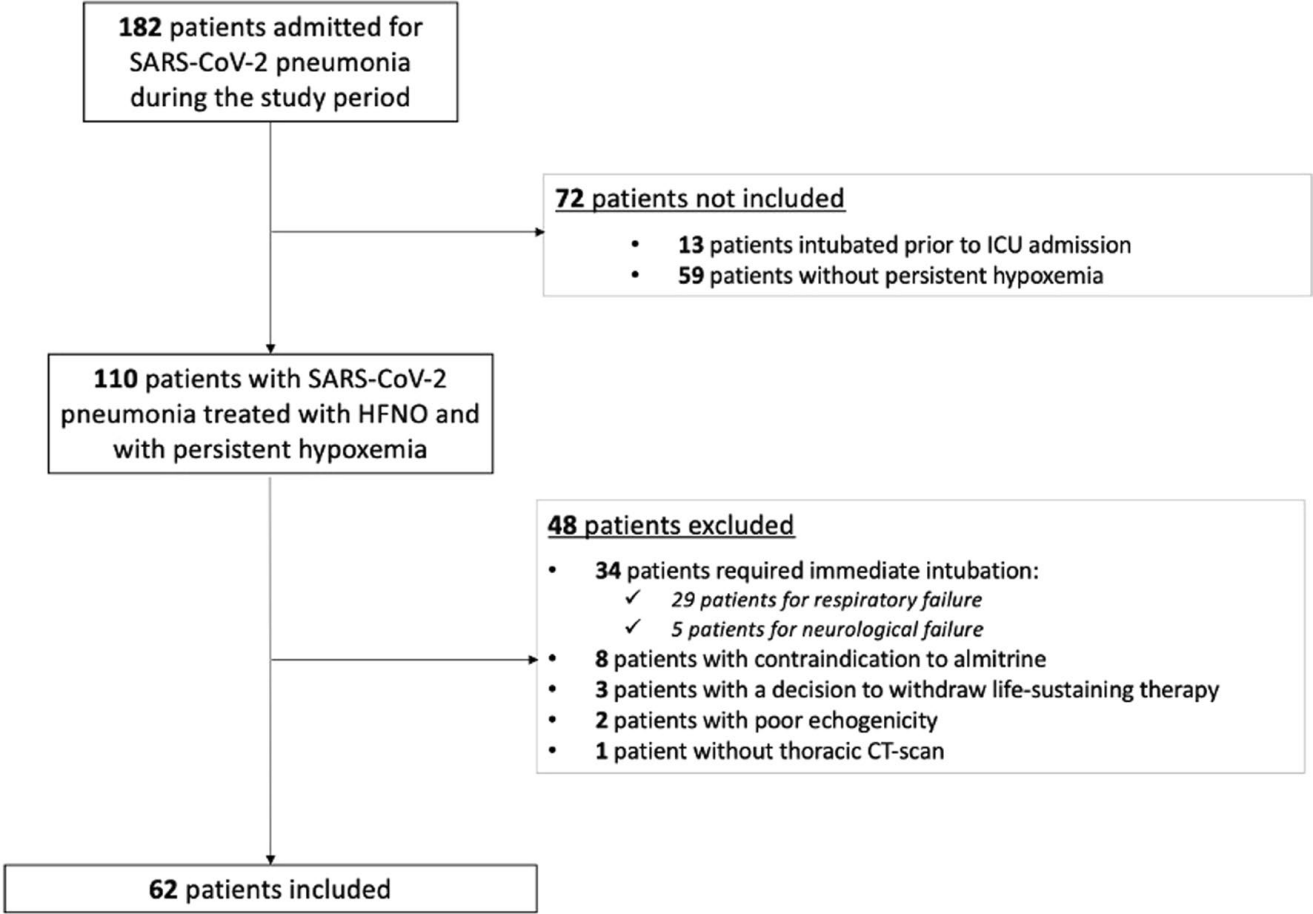
Flow chart of the study. HFNO: high-flow nasal cannula oxygen therapy, ICU: intensive care unit

Among the 182 consecutive patients admitted to our ICU for SARS-COV-2 pneumonia during the study period, 62 were included (Fig. 1): 41 (66%) were male, 20 (32%) had arterial hypertension, 13 (21%) had diabetes mellitus, 28 (45%) had obesity and 2 (3%) patients were vaccinated against SARS-CoV-2 (Table 1). The delay from the onset of symptoms to ICU admission was 9 (7–10) days, corticosteroids and tocilizumab were respectively administered in 62 (100%) and 55 (89%) patients and the ICU mortality rate was 10% (Table 1).

**Table 1:** Patient characteristics and outcomes according to the response to almitrine administration

	Non-responders (n = 16)	Responders (n = 46)	p value
Characteristics			
Age (years)	67 [56–71]	62 [53–67]	0.14
Male gender, n (%)	12 (75%)	29 (63%)	0.38
BMI, (kg/m^2^)	30 [27–33]	28 [25–34]	0.66
SAPS-2 score	40 [32–50]	31 [25–41]	0.01
SOFA score at ICU admission	2 [2–3]	2 [2–2]	0.30
Vaccination, n (%)	1 (6%)	1 (2%)	0.45
Arterial hypertension, n (%)	6 (38%)	14 (30%)	0.60
Diabetes mellitus, n (%)	3 (19%)	10 (22%)	1.00
Obesity, n (%)	8 (50%)	20 (43%)	0.65
Dyslipidemia, n (%)	4 (25%)	12 (26%)	1.00
Smokers, n (%)	1 (6%)	3 (6%)	1.00
Coronary artery disease, n (%)	1 (6%)	1 (2%)	0.45
Chronic heart failure, n (%)	0 (0%)	4 (9%)	0.56
Stroke, n (%)	1 (6%)	1 (2%)	0.45
Chronic respiratory disease, n (%)	3 (19%)	1 (2%)	0.04
Chronic kidney disease, n (%)	0 (0%)	1 (2%)	1.00
Immunosuppression, n (%)	0 (0%)	5 (11%)	0.32
Oxygenation and biological variable at ICU admission			
FiO_2_ (%)	85 [78–100]	80 [71–100]	0.86
PaO_2_ (mmHg)	61 [58–70]	65 [61–75]	0.17
PaO_2_/FiO_2_ ratio	74 [64–85]	82 [70–100]	0.19
SaO_2_ (%)	92 [91–96]	95 [93–96]	0.14
PaCO_2_ (mmHg)	32[31–35]	34[31–37]	0.85
Lactate (mmol/L)	1.2 [1.1–1.4]	1.1 [0.8–1.7]	0.52
Neutrophils count (× 10^9^/L)	7.8 [5.0–9.0]	6.3 [4.4–8.5]	0.52
Lymphocyte count (× 10^9^/L)	0.7 [0.6–0.9]	0.6 [0.4–0.8]	0.29
C-reactive protein (mg/L)	124 [50–189]	108 [64–172]	0.84
D-dimers (μg/L)	778 [604–1080]	757 [434–1244]	0.6
Fibrinogen (g/L)	6.0 [3.8–7.6]	5.8 [5.1–7.0]	0.63
Creatinine ( mol/L)	62 [52- 70]	62 [56–83]	0.47
Creatinine clearance (mL/min/1.73m^2^)	101 [84–109]	95 [87–104]	0.51
Treatments during ICU stay			
Dexamethasone, n (%)	16 (100%)	46 (100%)	1.00
Tocilizumab, n (%)	14 (88%)	41 (89%)	1.00
Norepinephrine, n (%)	10 (62%)	13 (28%)	0.01
Renal replacement therapy, n (%)	3 (19%)	0 (0%)	0.01
Delays and outcomes			
Delay from onset of symptoms to hospital admission (days)	8 [6–9]	7 [6–9]	0.77
Delay from onset of symptoms to ICU admission (days)	10 [8–10]	9 [7–10]	0.79
Delay from onset of symptoms to almitrine administration (days)	10 [8–11]	10 [8–12]	0.88
Delay from thoracic CT-scan to almitrine administration (days)	2 [1–2]	2 [1–3]	0.60
Delay from ICU admission to almitrine administration (hours)	7 [1–14]	6 [1–33]	0.29
Delay from awake prone positioning to almitrine administration (hours)	4 [2–6]	3 [2–7]	0.48
Duration of awake prone positioning session (hours)	2 [2–4]	3 [2–5]	0.09
Delay from ICU admission to intubation (hours)	17 [8–24]	23 [14–38]	0.13
Delay from almitrine administration to intubation ( hours)	6 [2–9]	25 [7–37]	< 0.01
Non-invasive ventilation, n (%)	0 (0%)	2 (4%)	1.00
Intubation, n (%)	14 (88%)	15 (33%)	< 0.01
Ventilator-free days at 28-Day (days)	19 [2–24]	28 [22–28]	< 0.01
ICU length of stay (days)	12 [7–30]	5 [3–10]	< 0.01
Hospital length of stay (days)	19 [13–25]	17 [9–30]	0.32
ICU mortality, n (%)	1 (6%)	5 (11%)	0.96
Mortality at 28-Day, n (%)	0 (0%)	4 (9%)	0.56
Mortality at 90-Day, n (%)	2 (7%)	5 (11%)	1.00

Variables are expressed as median [interquartile range] or number (percentages)

*FiO*_*2*_ inspired oxygen fraction; *ICU* intensive care unit; *PaO*_*2*_ partial arterial pressure of oxygen; *PaCO*_*2*_ partial arterial pressure of carbon dioxide; *SaO*_*2*_ arterial oxygenation saturation; *SAPS* simplified acute physiology score; *SOFA* sepsis-related organ failure assessment

### Effects of almitrine on oxygenation and respiratory variables

**Fig. 2:**
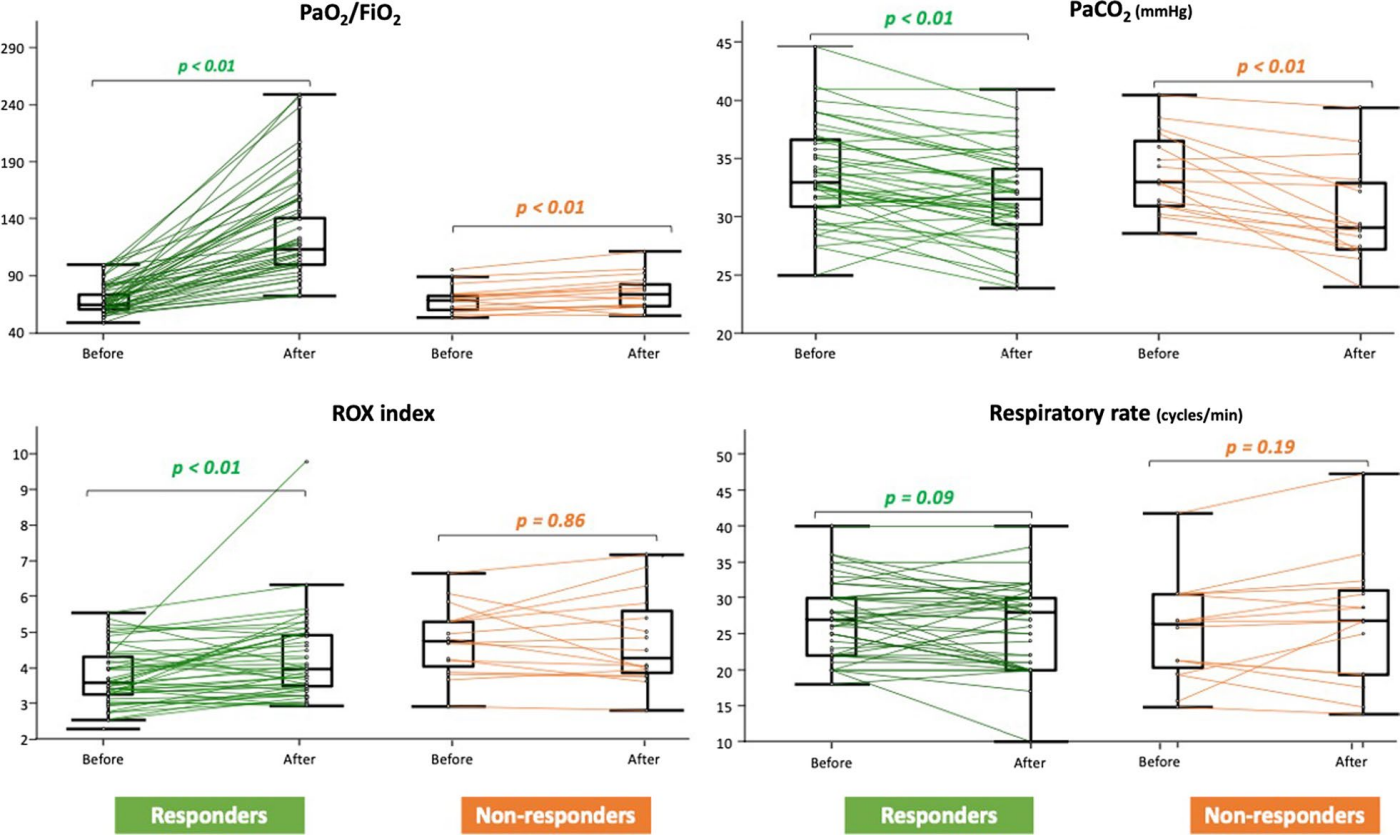
Effects of almitrine on respiratory and oxygenation variables (n = 62). The box shows the 25th and 75th percentiles, the line in the box the median and the whiskers the minimum and maximum values. Lines represent the individual changes (green lines for responders and orange lines for non-responders). FiO_2_: inspired oxygen fraction; PaO_2_: partial arterial pressure of oxygen, PaCO_2_: partial arterial pressure of carbon dioxide

In the whole population, almitrine significantly increased the PaO_2_/FiO_2_ ratio by 50% (p < 0.01), the PaO_2_ by 76% (p = 0.01), the arterial oxygenation saturation by 4% (p < 0.01), the SpO_2_ by 5% (p < 0.01) and significantly decreased the partial arterial pressure of carbon dioxide by 7% (p = 0.01) whereas the respiratory rate remained unchanged (Table 2, Fig. 2). The almitrine-induced percentage increase in PaO_2_/FiO_2_ ratio correlated with the delay from almitrine administration to ICU admission (r = 0.37, p < 0.01) and with the delay from almitrine administration to the end of the awake prone positioning session (r = 0.38, p < 0.01), but not with the duration of the awake prone positioning session (r = 0.14, p = 0.26).

**Table 2:** Effects of almitrine bolus on clinical, oxygenation and echocardiographic variables in responders and non-responders

	Non-responders (n = 16)			Responders (n = 46)		
	Before almitrine	After almitrine	p-value	Before almitrine	After almitrine	p-value
Clinical variables						
Respiratory rate (cycles/min)	27 [22–32]	28 [20–32]	0.19	27 [22–30]	28 [20–30]	0.09
SpO_2_ (%)	94 [91–94]	95 [92–96]	0.01	93 [90–95]	98 [97–99]^$^	< 0.01
Respiratory discomfort (mm)	30 [20–50]	30 [20–40]	0.13	40 [20–50]	30 [20–40]	0.02
ROX index	4.13 [3.43–4.75]	3.60 [3.18–4.97]	0.86	3.61 [3.26–4.34]	4.00 [3.50–4.97]	< 0.01
Heart rate (bpm)	85 [76–88]	80 [72–87]	0.39	79 [70–86]	74 [66–83]	< 0.01
Systolic arterial pressure ( mmHg)	134 [112–153]	139 [127–150]	0.91	129 [117–148]	130 [120–148]	0.42
Diastolic arterial pressure (mmHg)	64 [55–71]	61 [58–72]	0.87	64 [53–72]	65 [57–73]	0.47
Mean arterial pressure (mmHg)	90 [76–100]	90 [83–96]	0.78	86 [76–99]	89 [79–98]	0.68
Oxygenation variables						
FiO_2_ (%)	90 [84–100]	90 [89–100]	0.33	100 [90–100]	100 [86–100]	0.32
PaO_2_ (mmHg)	61 [57–68]	68 [64–73]	< 0.01	65 [58–69]	116 [99–159]^$^	< 0.01
PaO_2_/FiO_2_	70 [62–75]	76 [64–85]	< 0.01	67 [61–80]	121 [102–162]^$^	< 0.01
SaO_2_ (%)	93 [92–94]	95 [94–96]	< 0.01	94 [91–95]	99 [98- 99]^$^	< 0.01
PaCO_2_ (mmHg)	33 [31–36]	29 [27–33]	< 0.01	33 [31–37]	32 [30–34]	< 0.01
Lactate (mmol/L)	1.1 [0.9–1.6]	1.4 [1.0–1.6]	0.09	1.3 [1.0–1.4]	1.2 [1.1–1.5]	1.00
Echocardiographic variables						
RVEDA/LVEDA	0.54 [0.50–0.55]	0.56 [0.52–0.60]	< 0.01	0.54 [0.50–0.60]	0.56 [0.51–0.60]	0.03
TAPSE (mm)	22 [18–26]	22 [18–25]	0.55	22 [20–25]	21 [20–25]	0.50
Tricuspid S wave (cm/s)	14 [11–17]	13 [11–15]	0.25	14 [13–17]	15 [13–16]	0.67
VTI of the LV outflow tract ( cm)	23 [23–26]	22 [19–25]	0.06	21 [18–23]*	20 [17–25]	0.14
Left ventricular ejection fraction (%)	55 [48–59]	55 [46–62]	1.00	56 [47–60]	56 [45–61]	1.00

Variables are expressed as median [interquartile range]

*FiO*_*2*_ inspired oxygen fraction; *LVEDA* left ventricular end-diastolic area; *PaO*_*2*_ partial arterial pressure of oxygen; *PaCO*_*2*_ partial arterial pressure of carbon dioxide; *RVEDA* right ventricular end-diastolic area; *SaO*_*2*_ arterial oxygenation saturation; *SpO*_*2*_ pulse oximetry; *TAPSE* tricuspid annular plane systolic excursion; *VTI* velocity–time integral

^*^p < 0.05 non-responders vs. responders before almitrine and ^$^p < 0.05 non-responders vs. responders after almitrine

Among patients, 46(74%) were responders with a duration of continuous almitrine administration of 33 [22–49] hours and 1(25%) patient with chronic respiratory disease was responder. The duration of the single awake prone positioning session prior to almitrine administration was 3 [2–4] hours and did not differ between responders and non-responders (Table 1). In all patients, awake prone positioning session was stopped early because of poor tolerance. The delay from the onset of symptoms to almitrine administration (10 [8–12] vs. 10 [8–11] days, p = 0.88), from ICU admission to almitrine administration (6 [1–33] vs. 7 [1–14] h, p = 0.29) and from awake prone positioning and almitrine administration (3 [2–7] vs. 4 [2–6] hours, p = 0.48) did not differ between responders and non-responders (Table 1). Non-responders had a higher SAPS-2 score and had more frequently chronic respiratory disease than responders (Table 1). The other patient characteristics as well as respiratory and oxygenation variables immediately before almitrine administration were similar between responders and non-responders and are summarized in Tables 1 and 2. The almitrine-induced decrease in partial arterial pressure of carbon dioxide did not differ between responders and non-responders (8.2 [4.3–13.3] % *vs*. 6.7 [0.1–12.1] %, p = 0.26) (Table 2). In responders, Almitrine significantly decreased the respiratory discomfort of patients by 10% (p = 0.02) and increased the ROX index by 10% (p < 0.01) (Table 2, Additional file 2: Fig. S2). The ROX index could not predict the response to almitrine (area under curve of 0.607 (95% CI 0.474–0.728), p = 0.22) (Additional file 2: Fig. S2).

### Effects of almitrine on oxygenation according to CT-Scan patterns

**Fig. 3:**
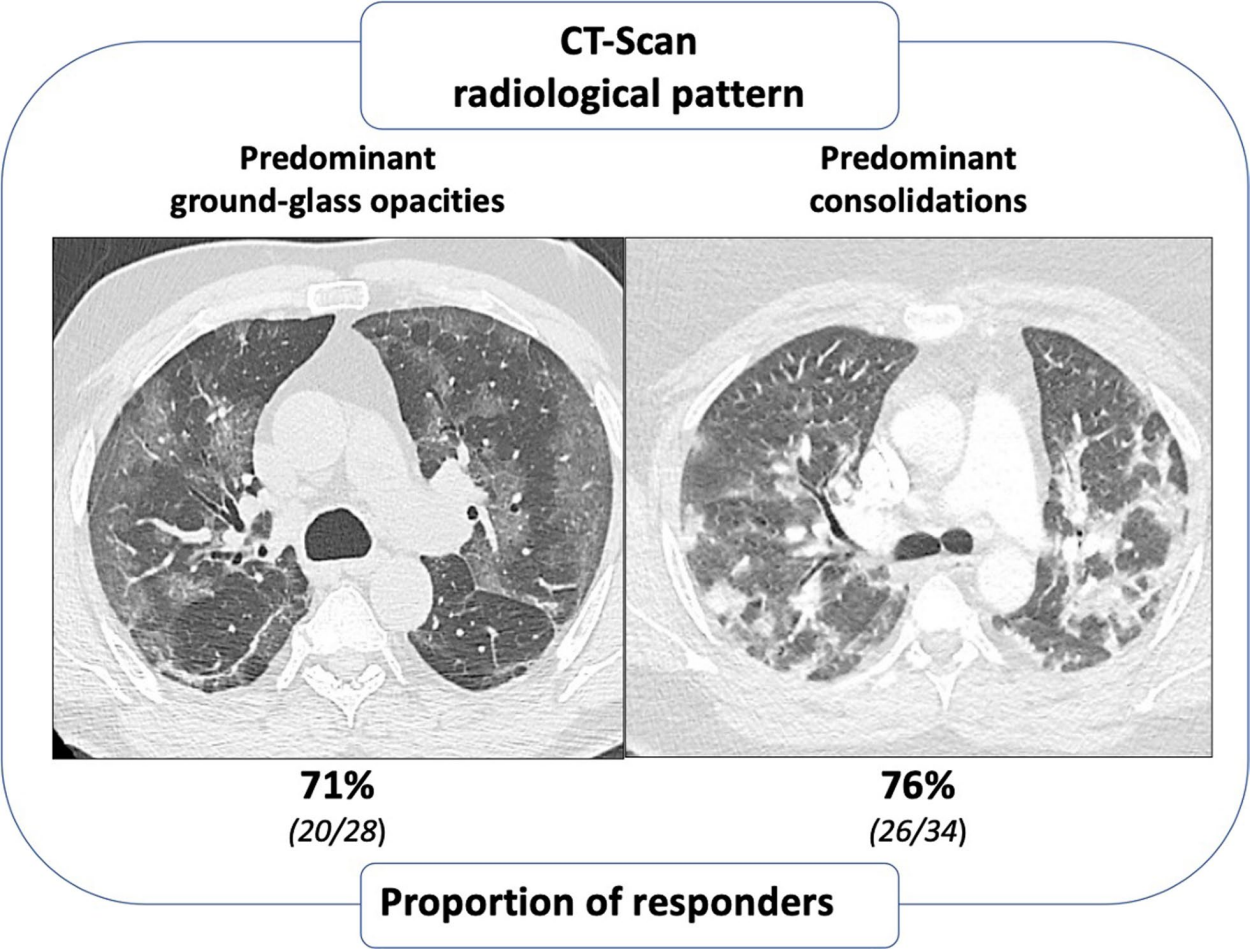
Proportion of responders according to the different thoracic CT-Scan radiological patterns

The delay from the onset of symptoms to thoracic CT-Scan (8 [6–9] vs. 7 [6–9] days, p = 0.50) and from thoracic CT-Scan to almitrine administration (2 [1–3] vs. 2 [1, 2] days, p = 0.60) did not differ between responders and non-responders (Table 1). Overall, 28 (45%) patients had predominant ground-glass opacities and 34 (55%) had predominant consolidations. All but one patient had vascular enlargement sign on thoracic CT-Scan. The number of areas with vascular enlargement sign as well as the other radiological variables did not differ between responders and non-responders (Table 3). The proportion of responders did not differ regardless of the CT-Scan pattern: 71% for the pattern with predominant ground-glass opacities and 76% for the pattern with predominant consolidations (p = 0.65) (Fig. 3).

**Table 3:** Thoracic CT-Scan analysis in responders and non-responders

	Non-responders (n = 16)		Responders (n = 46)	p-value
Extent of CT-Scan abnormalities (%)				0.15
< 10%	0 (0%)		0 (0%)	
10–25%	2 (12%)		9 (20%)	
25–50%	7 (44%)		12 (26%)	
50–75%	5 (31%)		24 (52%)	
> 75%	2 (12%)		1 (2%)	
Radiological patterns				0.65
Predominant ground-glass opacities, n (%)	8 (50%)		20 (43%)	
Predominant consolidations, n (%)	8 (50%)		26 (57%)	
Other radiological variables				
Number of areas with vascular enlargement sign	3 [2–5]		4 [2–5]	0.67
Number of areas with organizing pneumonia	2 [1–4]		4 [2–5]	0.14

Variables are expressed as median [interquartile range] or number (percentages)

### Intubation rate and other outcomes

**Fig. 4:**
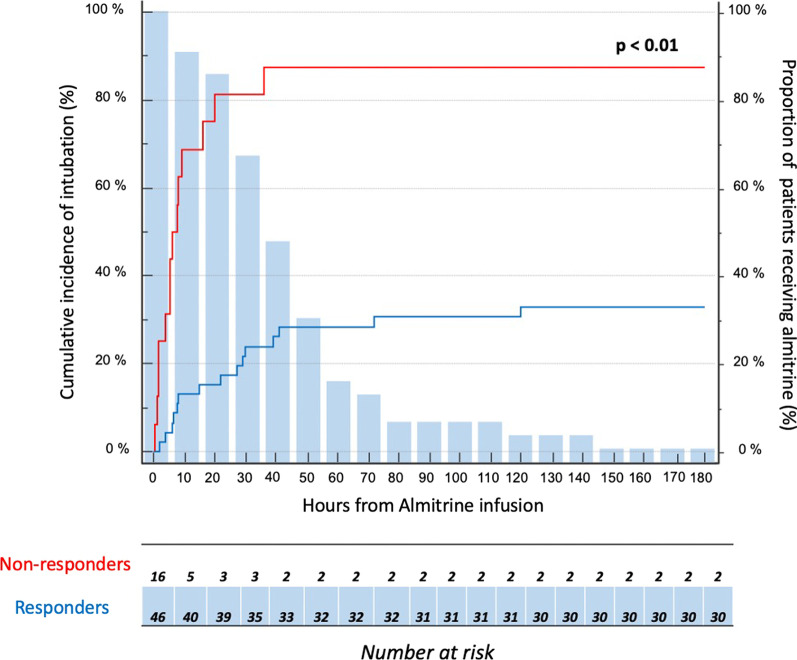
Cumulative incidence of intubation during the first week after almitrine administration in responders (red curve, n = 46) and non-responders (blue curve, n = 16) and proportion of patients receiving almitrine at each time point (blue bars)

The intubation rate was lower in responders than in non-responders (33 *vs.* 88%, p < 0.01). The cumulative incidence of intubation for the responders and non-responders is shown in Fig. 4. In both groups, all patients were intubated for respiratory failure only and the proportion of patients intubated for refractory hypoxemia did not differ between responders and non-responders (13% *vs.* 29% respectively, p = 0.39). No patient experienced pneumomediastinum or pneumothorax during their ICU stay. Responders tended to be intubated later than non-responders, but responders had more ventilator-free days at 28-day and a shorter ICU length of stay, while mortality rates in ICU, at 28-day and 90-day did not differ between the two groups (Table 2).

### Safety of almitrine

There was no difference in hemodynamic and echocardiographic variables between responders and non-responders before almitrine administration (Table 2). Almitrine bolus significantly increased the RVEDA/LVEDA ratio by 5% (p = 0.01), while hemodynamic and the other echocardiographic variables remained unchanged (Table 2). No patient experienced RV systolic dysfunction or acute cor pulmonale after almitrine bolus or during the whole duration of almitrine administration in responders. The incidence of venous toxicity was 21%.

## Discussion

So far, almitrine has been used as rescue therapy only in mechanically ventilated patients with COVID-19. In our study, three-quarters of spontaneously breathing patients with COVID-19 treated with HFNO and with persistent hypoxemia were responders to almitrine. The response to almitrine was not associated with any CT-Scan pattern and could not be predicted by the ROX index. Responders had a better respiratory comfort and a lower intubation rate than non-responders. Almitrine was well-tolerated and no patients experienced hemodynamic adverse effects.

To our knowledge, this is one of the first studies to assess the potential benefit of almitrine in spontaneously breathing patients with COVID-19. Like Muret and colleagues who previously reported improved oxygenation with almitrine in a spontaneously breathing patient with ARDS related to bacterial pneumonia [26], we found that almitrine improved oxygenation in spontaneously breathing patients with COVID-19 and persistent hypoxemia. We also found that 74% of patients were responders to almitrine, which is consistent with the proportion of responders found in studies including mechanically ventilated patients with ARDS related to COVID-19, in which almitrine was used as rescue therapy alone or in combination with inhaled nitric oxide [7–10]. Interestingly, the proportion of responders we found tended to be higher than that found in patients with ARDS not related to COVID-19 [22]. This may suggest the potential more marked impairment of the hypoxic pulmonary vasoconstriction in COVID-19 than in non-COVID-19 patients [4–6], as evidenced by (i) ventilation-perfusion mismatch with abnormal pulmonary vascular dilation and increased perfusion surrounding areas of lung opacity on dual-energy CT-Scan or subtraction CT imaging [27, 28] and (ii) lower pulmonary vascular resistance measured by pulmonary artery catheter in patients with COVID-19 [29].

We found that the almitrine-induced improvement in oxygenation was associated with a decrease in partial arterial pressure of carbon dioxide, while the patients’ respiratory rate remained unchanged. This may suggest that the improvement in oxygenation we observed with almitrine was partly related to a decrease in intrapulmonary shunt, secondary to an enhancement of hypoxic pulmonary vasoconstriction [30–34]. Other mechanisms are most likely involved to explain the effects of almitrine on oxygenation, as the almitrine-induced decrease in partial arterial pressure of carbon dioxide did not differ between responders and non-responders. Nevertheless, two previous studies in mechanically ventilated patients with COVID-19 found that almitrine alone [8] or in combination with inhaled nitric oxide [7, 8] improved oxygenation, unlike inhaled nitric oxide alone, highlighting that blunted hypoxic pulmonary vasoconstriction is likely one of the main mechanisms of ventilation-perfusion mismatch and hypoxemia in these patients. Although the impairment of hypoxic pulmonary vasoconstriction should therefore theoretically be more marked in responders than in non-responders, the RVEDA/LVEDA ratio, an indirect surrogate of pulmonary hypertension, did not differ before almitrine administration between the two groups of patients. It cannot be ruled out that some of the observed respiratory effects, such as the unchanged respiratory rate after almitrine administration in responders, may be partly related to a possible impairment of the central nervous system reported in critically ill patients with COVID-19 [35].

The radiological pattern on the thoracic CT-Scan performed prior to ICU admission was not predictive of the patients’ response to almitrine in terms of oxygenation. The proportion of responders was similar between patients with predominant ground-glass opacities and those with predominant consolidations. Moreover, the number of areas with vascular enlargement sign did not differ between responders and non-responders. Given the increased perfusion surrounding the peripheral areas of consolidations in patients with COVID-19 [27, 28] and that almitrine is a selective pulmonary vasoconstrictor in non-aerated areas, we initially hypothesized that the proportion of responders would be higher in patients with a radiological pattern with predominant consolidations and a high number of areas with vascular enlargement sign [27]. Our hypotheses to explain our results are as follows. First, in patients with COVID-19, hypoxic pulmonary vasoconstriction might be blunted in the same extent regardless of the type of CT-scan abnormalities, resulting in a marked intrapulmonary shunt even in patients with a radiological pattern with predominant ground-glass opacities. Second, some areas of consolidation could be related to pulmonary infarction, which would make almitrine inefficient in some patients with a radiological pattern with predominant consolidations, as areas of pulmonary infarction are not associated with increased perfusion [27]. Third, the pathophysiological mechanisms of vascular enlargement sign are not yet fully elucidated [20] and such radiological sign may therefore lack the specificity to be predictive of the response to almitrine. Further studies investigating lung perfusion during almitrine administration are needed to confirm these hypotheses and adequately address this question.

Interestingly, no patient experienced severe hemodynamic effects (RV systolic dysfunction or acute cor pulmonale), confirming the hemodynamic safety of almitrine previously evidenced by using invasive hemodynamic measurements in patients with ARDS related to COVID-19 [7, 10] or not [22]. We also found that responders had a lower intubation rate, more ventilator-free days at 28-day and a shorter ICU length of stay than non-responders, while the mortality rate did not differ between the two groups of patients. All these secondary exploratory results should be interpreted with caution as the study was neither designed nor powered for this purpose. The lower intubation rate may be explained by a more marked improvement in oxygenation and a better respiratory comfort, even if a recent randomized trial found that low-dose almitrine failed in reducing the need for intubation [36]. However, patients included in this study were less severe than those we considered and an eightfold lower dose of almitrine was administered [36]. The low mortality rate we found in both groups compared to the existing literature [37] may be explained by the fact that our patients were selected and less severe, as patients who required immediate intubation were excluded. Furthermore, we included patients from the second and third pandemic waves and not from the first wave and it cannot be excluded that different variants of SARS-CoV-2 and improved patient management may partly explain this discrepancy.

We acknowledge some limitations to our study. First, this was a single-center study. However, this implied that management of patients was homogeneous. Second, as the patients were treated with HFNO, the intrapulmonary shunt could not be calculated to confirm the physiological mechanisms involved in the almitrine-induced improvement in oxygenation. Third, it cannot be excluded that the radiological pattern at the time of almitrine administration differed from the CT-Scan performed prior to ICU admission, as CT-Scan was not repeated for obvious ethical reason. However, there was a short delay between CT-Scan and almitrine administration, which was similar in both groups of patients. Fourth, late effects of prone positioning on oxygenation cannot be excluded but the delay between awake prone positioning and almitrine administration was similar in both groups of patients. Fifth, no invasive monitoring of the right ventricle with a pulmonary artery catheter was available to confirm that responders might have a more marked impairment of hypoxic pulmonary vasoconstriction than non-responders. Sixth, we did not evaluate patients’ diaphragmatic function and the occurrence of patient self-inflicted lung injury during ICU stay. Seventh, non-responders had a higher SAPS-2 score and had more frequently chronic respiratory disease than responders, which could be a bias and therefore theoretically influence some of the outcomes. Finally, our study was neither designed (no control group and no blinding) nor powered to assess the effects of almitrine on intubation rate, duration of mechanical ventilation, ICU length of stay and mortality.

## Conclusions

Almitrine could be an interesting therapy in spontaneously breathing patients with COVID-19 treated with HFNO and with persistent hypoxemia, given its effects on oxygenation without serious adverse effects regardless of the CT-Scan pattern, and potentially on intubation rate. These preliminary results need to be confirmed by further randomized studies.

## Supplementary Information

**Additional file 1:** STROBE Statement checklist for the manuscript.

**Additional file 2:** Transthoracic echocardiographic measurements. **Figure S1.** Study protocol. HFNO: high-flow nasal cannula oxygen therapy, FiO_2_: inspired oxygen fraction; PaO_2_: partial arterial pressure of oxygen. **Figure S2.** Receiver operating characteristics curve for the ability of the ROX index to predict the response to almitrine (n = 62). AUC = area under the curve, expressed as mean [95% confidence interval].

## Data Availability

The datasets used and/or analyzed during the current study are available from the corresponding author on reasonable request.

## Abbreviations

ARDS: Acute respiratory distress syndrome
CI: Confidence interval
COVID-19: Coronavirus disease 19
FiO_2_: Inspired oxygen fraction
ICU: Intensive care unit
LVEDA: Left ventricular end-diastolic area
HFNO: High-flow nasal cannula oxygen therapy
PaO_2_: Partial arterial pressure of oxygen
RVEDA: Right ventricular end-diastolic area
SpO_2_: Pulse oximetry
