# Supplementary material for: Use of almitrine in spontaneously breathing patients with COVID-19 treated with high-flow nasal cannula oxygen therapy and with persistent hypoxemia
Source: Respir Res. 2023 Jan 5;24:1. doi: 10.1186/s12931-022-02308-y (PMC9812745; doi:10.1186/s12931-022-02308-y)
Supplement: Supplementary file 2 — Additional file 2: Transthoracic echocardiographic measurements. Figure S1. Study protocol. HFNO: high-flow nasal cannula oxygen therapy, FiO2: inspired oxygen fraction; PaO2: partial arterial pressure of oxygen. Figure S2. Receiver operating characteristics curve for the ability of the ROX index to predict the response to almitrine (n = 62). AUC = area under the curve, expressed as mean [95% confidence interval]. [file 12931_2022_2308_MOESM2_ESM.docx]

**Additional file 2**

All transthoracic echocardiographic measurements were performed with a Vivid 9 (GE, Healthcare, Chicago, Il, USA) by the same experienced operator (CS). All measurements were performed at end-expiration and averaged on three consecutive cardiac cycles in patients with sinus rhythm and on five consecutive cardiac cycles in patients with atrial fibrillation, according to current recommendations (1, 2). From apical five- and four-chamber views, we measured (i) the velocity-time integral of the left ventricular outflow tract, (ii) parameters of right ventricular systolic function with the systolic peak velocity of the tricuspid annulus with tissue Doppler imaging and the tricuspid annular plane systolic excursion in the M-mode, and (iii) the left and right ventricular end-diastolic areas (LVEDA and RVEDA). All contours were hand-drawn. The left ventricular ejection fraction was calculated by the modified Simpson’s rule, and we calculated the RVEDA/LVEDA ratio. Acute cor pulmonale was defined by a RVEDA/LVEDA ratio >0.6 and flattened interventricular septum (1).

**Figure S1**


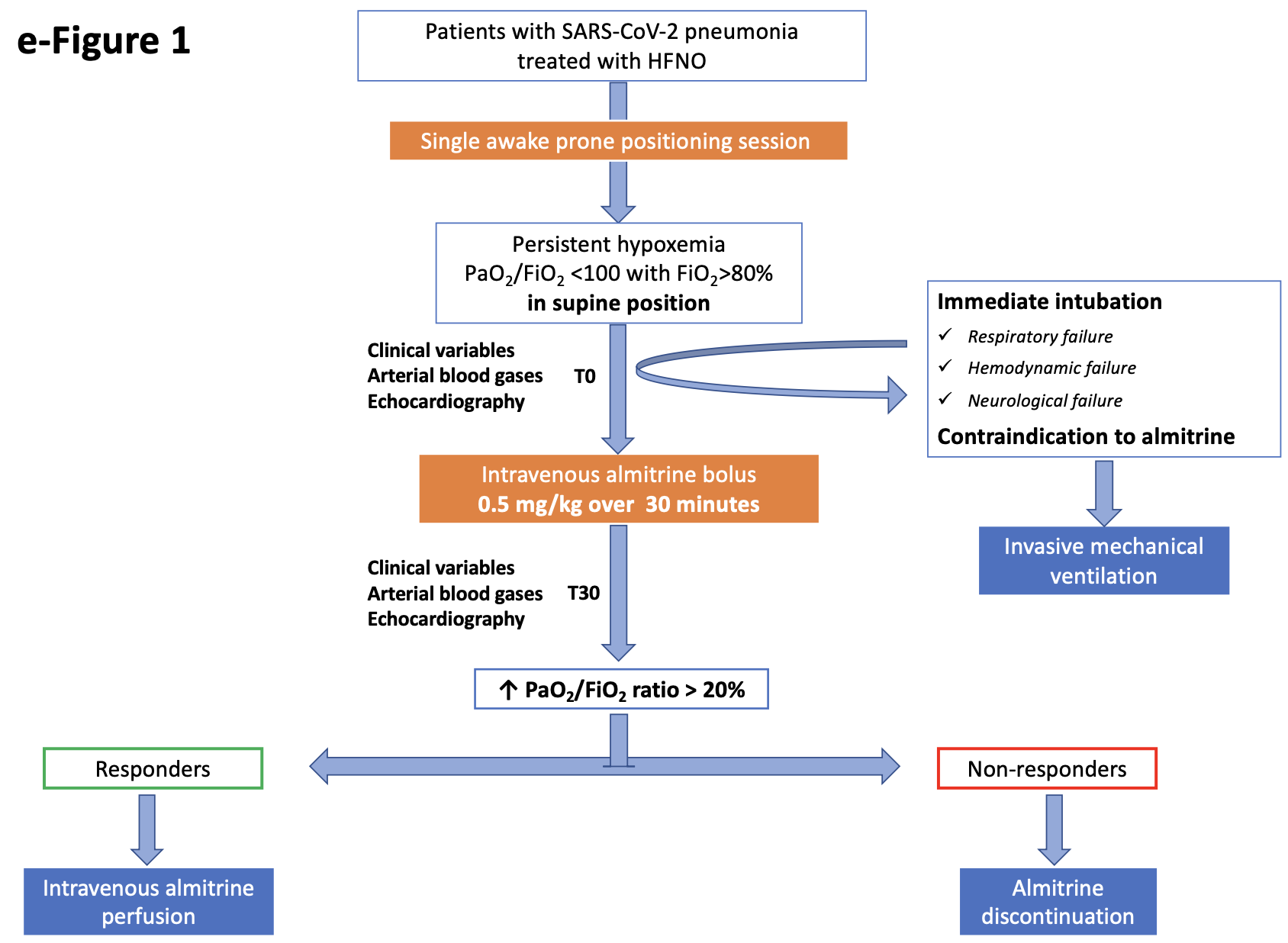


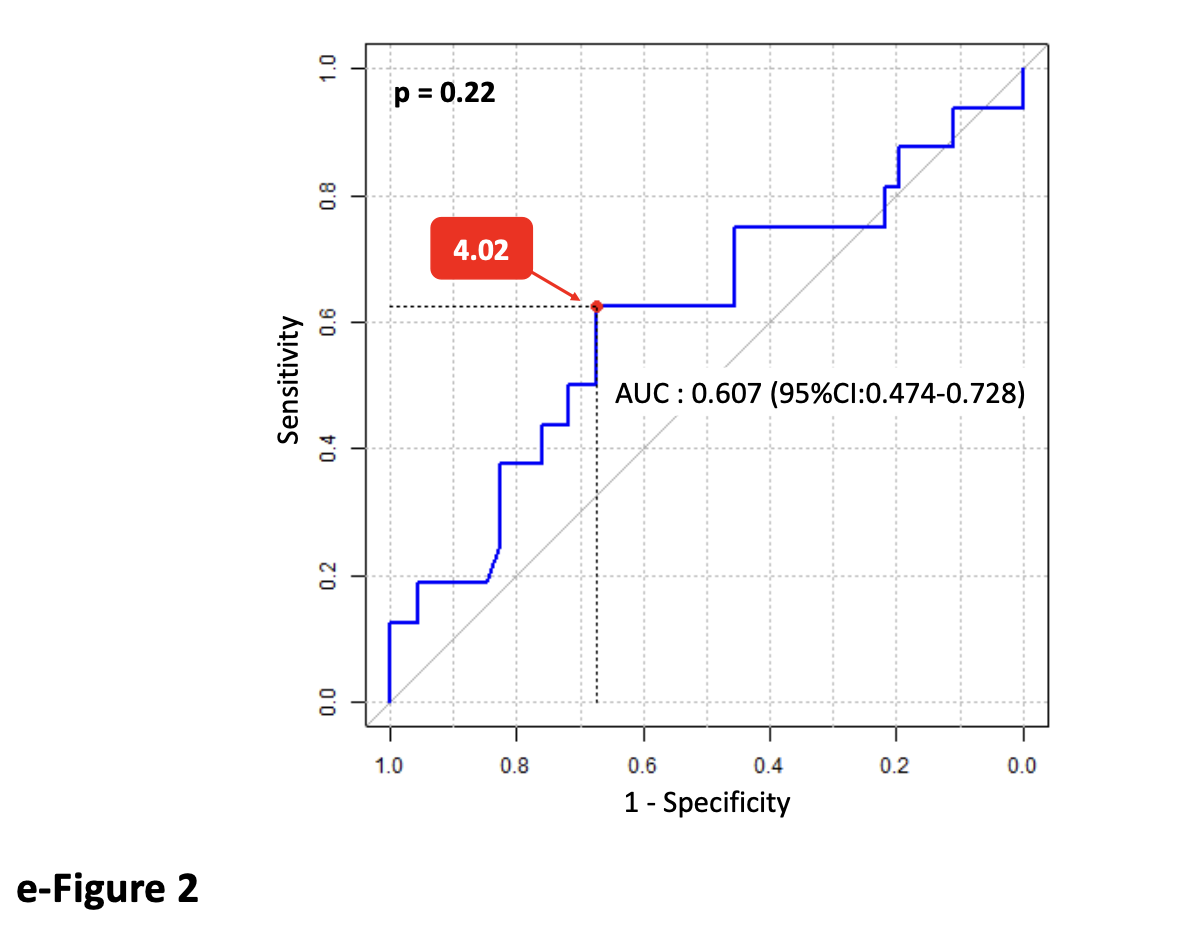
**Figure S2**

**Additional references**

1. Lang RM, Badano LP, Mor-Avi V, Afilalo J, Armstrong A, Ernande L, et al. Recommendations for cardiac chamber quantification by echocardiography in adults: an update from the American Society of Echocardiography and the European Association of Cardiovascular Imaging. Eur Heart J Cardiovasc Imaging. 2015;16(3):233-70.

2. Mitchell C, Rahko PS, Blauwet LA, Canaday B, Finstuen JA, Foster MC, et al. Guidelines for Performing a Comprehensive Transthoracic Echocardiographic Examination in Adults: Recommendations from the American Society of Echocardiography. J Am Soc Echocardiogr. 2019;32(1):1-64.
